# The effect of a curriculum-based physical activity intervention on accelerometer-assessed physical activity in schoolchildren: A non-randomised mixed methods controlled before-and-after study

**DOI:** 10.1371/journal.pone.0225997

**Published:** 2019-12-05

**Authors:** Alison L. Innerd, Liane B. Azevedo, Alan M. Batterham

**Affiliations:** School of Health and Life Sciences, Teesside University, Middlesbrough, United Kingdom; University of New Mexico, UNITED STATES

## Abstract

Classroom-based physical activity (PA) interventions offer the opportunity to increase PA without disrupting the curriculum. We aimed to explore the feasibility and potential effectiveness of a classroom-based intervention on moderate to vigorous PA (MVPA) and total PA. The secondary aim was to assess the acceptability and sustainability of the intervention. In a mixed-methods, non-randomised, exploratory controlled before-and-after study, 152 children (10 ± 0.7 years) were recruited from five schools; two intervention (n = 72) and three control (n = 80) schools. School teachers delivered an 8-week classroom-based intervention, comprising of 10 minutes daily MVPA integrated into the curriculum. The control schools maintained their usual school routine. Mean daily MVPA (min), total PA (mean cpm), physical fitness, and health-related quality of life measurements were taken at baseline, end of intervention, and 4-weeks post-intervention (follow-up). Data were analysed using a constrained baseline longitudinal analysis model accounting for the hierarchical data structure. For the primary outcomes (MVPA and total PA) the posterior mean difference and 95% compatibility interval were derived using a semi-Bayesian approach with an explicit prior. The acceptability and sustainability of the intervention was explored via thematic content analysis of focus group discussions with teachers (n = 5) and children (n = 50). The difference in mean daily MVPA (intervention-control) was 2.8 (-12.5 to 18.0) min/day at 8 weeks and 7.0 (-8.8 to 22.8) min/day at follow-up. For total PA, the differences were -2 (-127 to 124) cpm at 8-weeks and 11 (-121 to 143) cpm at follow-up. The interval estimates indicate that meaningful mean effects (both positive and negative) as well as trivial effects are reasonably compatible with the data and design. The intervention was received positively with continuation reported by the teachers and children. Classroom-based PA could hold promise for increasing average daily MVPA, but a large cluster randomised controlled trial is required.

## Introduction

The benefits of physical activity (PA) to children’s health are well established. However, in England only 23% of boys and 20% of girls meets the UK Chief Medical Officers’ recommendation of at least 60 min of at least moderate intensity physical activity per day [1], showing the need to explore different strategies to increase daily PA in children. The school setting is an obvious choice for implementation of health promotion initiatives, including PA, due to children spending most of their waking weekday hours at school [2]. There is a strong body of evidence suggesting that school-based interventions are effective to increase physical activity, and to a lesser extent fitness, in schoolchildren, at least in the short-term [3, 4]. However, there are still inconsistencies concerning the effectiveness of school-based and multicomponent interventions and a lack of high-quality intervention studies [5, 6].

Several PA interventions have been implemented in schools to promote PA including playground interventions [7], walking schemes and active travel [8], and extra-curricular activities [9, 10]. Designing school-based PA interventions can be challenging, as the intervention should be incorporated into the school environment effectively and be easy to deliver and maintain by the school. However, factors such as time (competing requirements, teacher overload), resource availability, and supportive school climate might affect implementation [2]. Schools are also under academic performance pressure, which often results in a reduction of Physical Education time and PA opportunities to allow time to meet the academic objectives [11]. Therefore, a successful PA intervention should be fully integrated into the curriculum. A number of classroom-based PA interventions have been delivered by schoolteachers including: Physical Activity Across the Curriculum (PAAC) [12, 13], Energisers [14]), ‘Active Classrooms’ [15] and Take 10! [16, 17]. These studies found that curriculum-based activities may promote PA [13, 14, 15, 16], increase time on task [13, 14], and might improve academic performance [18] in schoolchildren. However, these studies were implemented in the US and Ireland and none to our knowledge were delivered in the UK. Likewise, there is little evidence on teachers’ views of these curriculum-embedded interventions. One study investigated teacher enjoyment in classroom-based activities [19]; however, enjoyment was only measured by direct observations by the researcher, which can bias the output. Teachers are receptive to delivering activity breaks in the classroom in isolation to academic content [20]. Nevertheless, little is known about how the teachers deliver curriculum-based activities in schools and the challenges that they face, which could affect future interventions. Similarly, there are no studies reporting children’s views on classroom-based interventions.

The primary aim of this study was to test the feasibility and potential effect of a classroom-based, curriculum-embedded intervention—ExCiTE; Exercise Classes in the Teaching Environment—on total PA and moderate-to-vigorous physical activity (MVPA). The secondary aim was to measure and describe health-related quality of life and physical fitness components, and to gain understanding of the experiences, views, and attitudes towards the ExCiTE intervention among participating children and the schoolteachers.

## Methods

### Study design

This was a non-randomised, exploratory, controlled before-and-after design with a mixed-methods approach. The study took place from and January 2011 to July 2011. The protocol was registered retrospectively on clinicaltrials.gov (trial number NCT04119076) in October 2019. When the planned randomised trial was changed to a non-randomised (observational) study (see below), incorrectly we did not view prospective registration as a requirement. The design, conduct, and reporting of the trial adheres to the Transparent Reporting of Evaluations with Non-randomised Designs statement [21](S2 File). Mainstream primary schools within the same Local Authority in the North East of England were invited via email to participate. In the UK, a Local Authority is an organization that is officially responsible for all the public services and facilities in a particular geographical area. All 22 primary schools in the Local Authority were contacted. Initially, Head Teachers approached at two schools (school 1 and school 5) expressed an interest to take part but stated that they would not consent to be randomised, citing competition for school resource use at that time (preferring control allocation) or preference for allocation to the intervention. Consequently, we adapted the study by employing a non-randomised controlled before-and-after design. These two schools were scored by a proxy for average socio-economic background of the students: Index of Multiple Deprivation (IMD). Schools that closely matched the two initial schools for IMD were approached to take part in the study via an email. If a school declined, another matching school within the range (IMD 0–10,000; 10,000–20,000; > 20,000) was approached. We used the proportion of students eligible for free school meals as a secondary marker for socio-economic background. The final sample included five primary schools; two schools agreed to the Intervention condition and three to the Control, based on the initial school that they matched. The study was approved by School of Health and Social Care Research Ethics sub-Committee at Teesside University (Protocol No 209/10). Prior to data collection (objective data collection and focus groups), parents provided written informed consent for their child to take part in the study. Written and verbal child assent was obtained prior to all data collection. Teachers who taught Years 5 and Year 6 in the intervention schools were given a participant information sheet explaining the purpose of the study and provided written consent.

### School demographics

The details of the five schools recruited are displayed in Table 1

**Table 1:** School demographic.

	Condition	FSM eligibility	IMD	Total number of students at school
School 1	Intervention	5.2%	26,666	302
School 2	Intervention	37.8%	8,845	230
School 3	Control	0%	28,501	296
School 4	Control	28.6%	9, 434	241
School 5	Control	19.7%	5, 235	220

FSM = Free School Meals. IMD = Index of Multiple Deprivation

### Participants

All children in Years 5 and 6 (age range 9–11 years) from five schools (n = 195) were invited to take part in the study.

### ExCiTE Intervention

An activity and resource pack were developed based on previously developed programmes and following consultations with primary school teachers. The activity and resource packs were paper-based booklets. The resource pack explained the purpose of the intervention, offered teaching tips for movement tasks to deliver (e.g. jumping jacks) and suggestions for classroom layout. The activity pack contained examples of activities from a range of curriculum subjects (Maths, English, Science, Music and Humanities). The examples gave clear instructions for the teacher to deliver academic content actively, such as Jumping Maths where the teacher calls out maths challenges and the students must answer by performing the required number of jumps (S4 File). A pilot study was conducted to determine the intensity of the ExCiTE activities using indirect calorimetry and a scoring system was then created based on the measured intensity. The intensity and level of preparation required was indicated on the activity examples, so it was clear for the teachers to select an activity based on the presumed energy levels of the class and preparation required.

All classes in Year 5 and Year 6 at the Intervention schools (N = 2) were selected. In one of the schools this consisted of three classrooms and for the other, two classrooms. Each class was taught by one teacher, and the same teacher throughout the day. Schoolteachers were asked to deliver at least one activity from the activity pack daily for 8 weeks. Schoolteachers were asked to note the activity, date, duration, subject area, and an indication of how well the session went in a log diary. Each activity lasted approximately 10-min and the teachers were requested to deliver one activity on each school day at a time to suit the lesson plan. Children in control schools maintained their usual school routine activities. There were two classes in control school 3 and one class in schools 4 and 5.

The teachers were given a one-hour training session by the lead author (AI) who explained and demonstrated how to implement the intervention activities. The training consisted of the lead author demonstrating the different movements the teachers could deliver, explaining the structure and layout of the activity pack and the log diary, and answering any questions from the teachers. To support the implementation of the intervention, several procedures were put in place. Firstly, the teachers received weekly reminders by the lead author. Secondly, the teachers were encouraged to complete a daily log of when the activities were implemented. Finally, the teachers were observed by the lead author (AI) mid-way through the intervention and they were given a questionnaire twice (midway and end) to determine the quality of the delivery. The quality was assessed using teacher self-assessment and through questions and answers with the observer.

### Outcome measures

Measurements were taken at baseline, post-intervention (after the intervention) and follow up (4 weeks after the conclusion of the intervention).

#### Anthropometrics

Height was measured to the nearest 0.1 cm using a portable stadiometer (Leicester Height Measure, Child Growth Foundation, London, United Kingdom). Weight was measured to the nearest 0.1 kg using calibrated scales (Seca 761, Seca Weighing and Measuring Systems, Birmingham, England). The measurements were taken in a private area in the classroom or sports hall, children wore light clothing, and shoes were removed.

#### Physical activity measurement

Mean daily total PA and MVPA (primary outcomes) were measured using a hip-mounted accelerometer (ActiGraph GT3X). To detect the intermittent and sporadic nature of child activity, data were recorded in 10-s epochs. The children were instructed to wear the activity monitors during waking hours for seven full consecutive days, which has shown be sufficient to reliably estimate habitual physical activity [22]. The children and teachers were given clear written and verbal instructions as to how to wear the monitor. The children were also instructed to keep a log on when they put the monitors on and took them off. ‘Non-wear time’ classification was determined by a period of 20 consecutive minutes with zero accelerometer counts [23]. Data was processed using the ActiLife Software (version 6.13.4) for days which contained at least 10 hours of wear time. To be included in the analysis the children must have worn the monitor for at least four valid days. This criterion is in line with previous research with children and adolescents [24]. Daily total PA using the vertical axis data was reported in mean count per minute (cpm). MVPA was estimated using the Evenson cut-point for moderate intensity physical activity [25]). Given positive correlations between wear time and MVPA, and differences in wear time between intervention and control, MVPA was adjusted for wear time by including wear time as a covariate in the analysis model. For total PA, average counts per minute is uncorrelated with wear time as the variable is derived as total counts across the valid days divided by total wear time. Processing of the accelerometry data to derive the primary outcome measures was not blinded to condition assignment.

#### Health–Related quality of life

In a quiet area, the children were asked to complete the 27-item Kidscreen Questionnaire [26]. This questionnaire assesses health-related quality of life across five dimensions: physical well-being, psychological well-being, parent relations and autonomy, social support and peers and school environment. The children answered each question on a 1–5 scale in relation to the intensity of the attitude (not at all, slightly, moderately, very, extremely) or the frequency (never, seldom, quite often, very often, always). All subscale scores were reported as t-values based on the Swiss community normative data, with a mean of 50 and a standard deviation of 10. The higher the score, the better the quality of life.

#### Aerobic fitness

Aerobic fitness was measured using the multistage fitness test, a twenty-metre shuttle-run test, using the British National Coaching Foundation protocol [27]. The test was performed outside on the school playground. Approximately ten children took part in the test at one time to ensure the test could be monitored accurately by the researchers. The running speed from the final shuttle level was expressed as the effective running speed (km/h). Level one of the multistage fitness test starts at 8.5km/h, increasing by 0.5km/h for each subsequent level, with a set number of shuttles per level. The effective running speed was calculated as the level running speed, plus the proportional increase in speed throughout the shuttle at the time of termination.

#### Physical fitness

A fun fitness test was developed to assess several components of fitness based on the Eurofit programme [28] including; flexibility (sit and reach test); balance (flamingo balance); speed (10 x 5m shuttle run), hand-eye coordination (plate tapping); explosive strength (broad jump); and muscular strength and endurance (sit ups). The children were given a verbal and visual demonstration before each activity and they were also given the opportunity to have a practice of each activity. The children completed the activities in pairs. The activities were undertaken in the following order as suggested by Eurofit: 1. Flamingo Balance test, 2. Plate Tapping, 3. Sit-and-Reach, 4. Standing Broad Jump, 5. Sit-Ups, 6. 10 X 5 m Shuttle Run.

#### Structured discussions & focus groups

Following the intervention, two structured discussions were conducted in the intervention schools, with five teachers and nine focus groups of five to six children. The structured interviews and focus groups took place at the four-week follow up timepoint. The primary purpose of the structured discussions with teachers was to determine the applicability and sustainability of the ExCiTE intervention and the purpose of the child focus groups was to assess enjoyment, the effect on learning, and suggestions for future developments.

### Statistical analysis

Data were analysed, as per the allocated conditions, using a constrained longitudinal baseline model [29] including fixed effects for intervention, sex, sex*intervention, and wear time, and random effects for cluster (school), individual nested within cluster, and time nested within cluster. A Satterthwaite correction of degrees of freedom was applied. This model accounts for the hierarchical data structure and provides a principled method for dealing with missing outcome data, including missing baseline data as baseline is part of the outcome vector in this linear mixed model [30]. Mean intervention effects at the 8-weeks and 3-months follow up (4-weeks post-intervention) timepoints are presented together with 95% posterior compatibility intervals. For the primary outcomes, the posterior distribution was derived by combining the observed results with an explicit prior distribution. This normal prior was centred on a mean treatment effect of zero with 95% limits of ± 40 min/day for MVPA or ± 250 for average counts per min for total PA, reflecting our belief that any treatment effect would not be extremely large (>2 SD). The mean and variance of this prior was combined with the observed mean (point estimate) and variance (SE^2^) for the intervention effect at 8-weeks and 3-months using information-weighted averaging [31], equivalent to a fixed-effects meta-analysis of the prior and the observed data. This method provides a point estimate of the intervention effect with appropriate shrinkage, together with a 95% posterior compatibility interval.

Using the obtained posterior distribution, the probability that the intervention effect would be above a minimum clinically important difference (MCID) of 5 min/day for MVPA was derived using the point estimate and combined SE from the information-weighted averaging using the Z distribution. The required Z score was computed as: (MCID-mean intervention effect)/combined SE, with the tail probability to the right of Z giving the probability that the intervention effect is >MCID. Qualitative terms were assigned to the derived probabilities using the following scale; <0.5%, most unlikely; 0.5 to 5%, very unlikely; 5 to 25%, unlikely; 25 to 75%, possibly; 75 to 95%, likely; 95 to 99.5%, very likely; >99.5%, most likely [32]. There is no robust clinical anchor for the MCID for MVPA in children. We define the MCID as the increase in MVPA that would be required to increase by 10% the proportion of children in the sample meeting the recommendation of an average of 60 min/day of MVPA; this increase was 5 min/day herein. There is no robust definition of the MCID for total physical activity (average counts), and we therefore simply present the point estimate and posterior compatibility interval for this variable.

For the secondary outcomes (fitness and quality of life variables), we present mean treatment effects together with 95% compatibility intervals from the same constrained longitudinal baseline analysis. These results are purely exploratory and descriptive. Therefore, no prior information is incorporated into the analysis of these outcomes. All analyses were performed using Stata software (StataCorp, 2017. Stata Statistical Software: Release 15, College Station, TX, USA: StataCorp LP).

### Structured discussion & focus group analysis

Following the focus groups with the children and structured discussion with the teachers, the data was transcribed by the lead author. The transcripts were analysed using the thematic content analysis method [33]. Following the ‘open-coding’ exercise, the different themes were placed into various categories. Due to the exploratory nature of this study, the thematic groupings were only reported, and we did not explore interconnectivity. We conducted participant checks with the teachers following the coding exercise to determine that the results represent what was said.

## Results

### ExCiTE intervention

**Fig 1:**
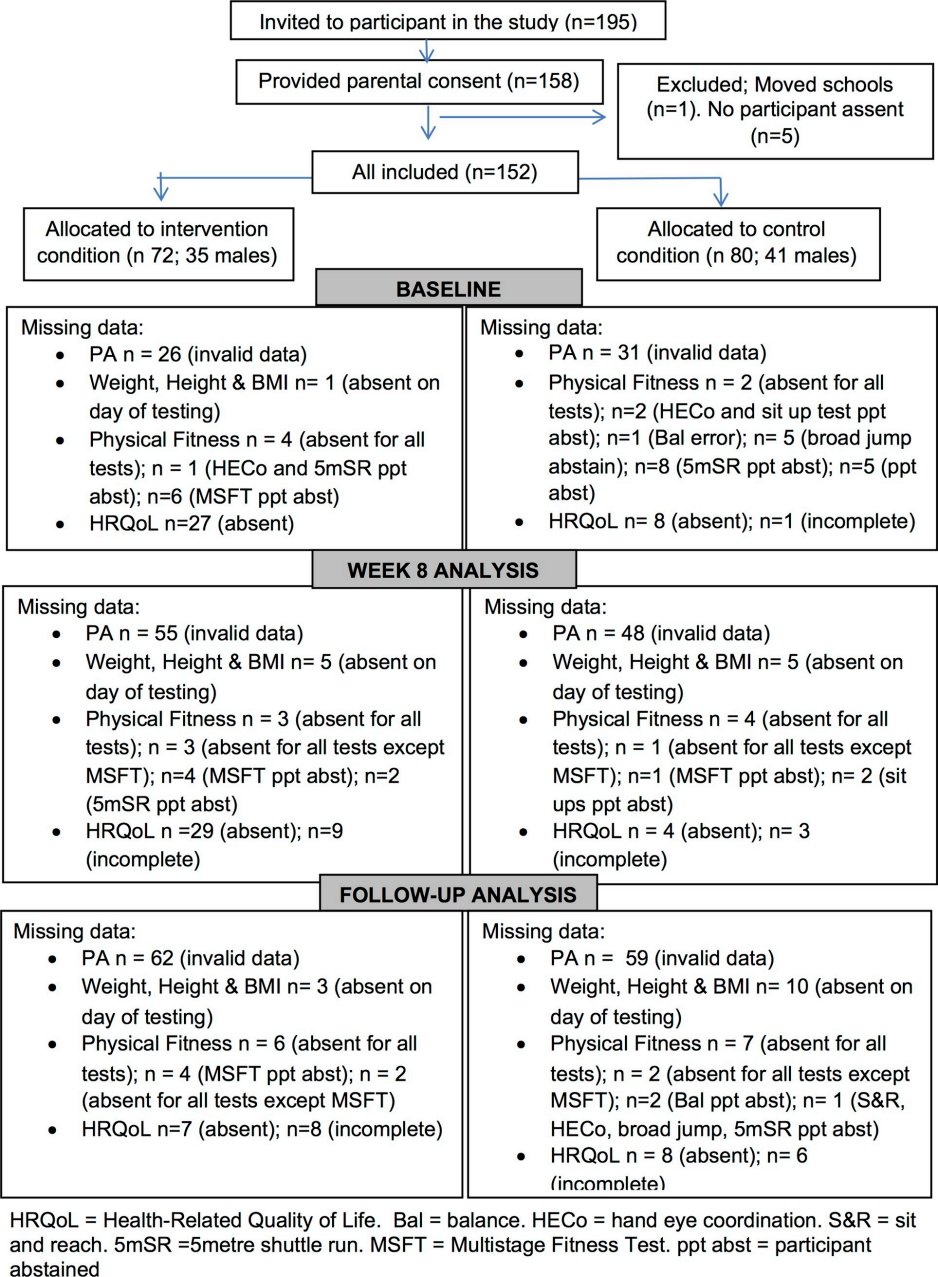
Flowchart of participants.

One-hundred and fifty-eight children provided parental consent and child assent; however, five children dropped out of the study prior to baseline measurements and one participant moved schools, giving the final sample of 152 (n = 76 boys), including 72 children from the Intervention schools and 80 from the Control schools. Participant uptake was 84% in the intervention school and 78% in the control school. Participant flow through the study is presented in Fig 1.

Five schoolteachers delivered the ExCiTE activities. On average, the intervention was delivered three times a week. Most of the activities were delivered in the morning, with Mathematics-based activities being the most commonly reported.

Sample characteristics at baseline are presented in Table 2.

**Table 2:** Sample baseline characteristics. Data presented as mean ± SD (N).

Variable		Intervention	Control
Descriptive	Height (cm)	142.1 ± 6.3 (71)	143.43 ± 6.6 (80)
	Mass (kg)	37.5 ± 9.6 (71)	37.9 ± 7.3 (80)
	BMI (kg/m^2^)	18.4 ± 3.6 (71)	18.3 ± 2.6 (80)
	Age (years)	9.9 ± 0.7 (72)	10.1 ± 0.7 (80)
Physical Activity	MVPA (min/day)	52.3 ± 16.4 (46)	63.1 ± 20.8 (49)
	Total PA (cpm)	503 ± 112 (46)	563 ± 126 (49)
Physical Fitness	Bal (no. of falls)	8.8 ± 3.9 (68)	6.4 ± 3.6 (77)
	HECo (s)	154.2 ± 21.2 (67)	143.2 ± 20.1 (76)
	S&R(cm)	17.3 ± 5.8 (68)	17.6 ± 8.2 (78)
	Broad Jump (cm)	129.3 ± 26.0 (68)	133.1 ± 22.8 (73)
	Sit Ups (no. completed)	14.7 ± 5.8 (68)	16.8 ± 4.7 (76)
	5m SR (s)	211.9 ± 25.4 (67)	207.1 ± 17.6 (70)
	ERSpeed (km/h)	10.1 ± 0.8 (62)	10.6 ± 0.9 (73)
HRQoL	Phys WB	53.6 ± 11.1 (45)	52.5 ± 10.1 (71)
	Psych WB	53.5 ± 12.2 (45)	51.1 ± 10.1 (71)
	Autonomy & PR	48.9 ± 13.2 (45)	48.3 ± 10.6 (71)
	Peer & SS	53.1 ± 12.8 (45)	50.9 ± 10.3 (71)
	School Enviro	53.9 ± 11.7 (45)	53.1 ± 9.9 (71)

HRQoL = Health-Related Quality of Life. Total MVPA = total moderate-to-vigorous physical activity. Total PA = daily total physical activity. Bal = balance. HECo = hand eye coordination. S&R = sit and reach. 5mSR = 5metre shuttle run. ERSpeed = effective running speed. Phys WB = physical wellbeing. Psycho WB = psychological wellbeing. Autonomy & PR–autonomy and parent relations. Peer & SS = peer and social support. School Enviro = school environment.

Missing PA data at baseline and follow-up were due to the participants not meeting the wear time criteria (4 days, 10 hours) and this compliance decreased across the testing phases for both groups. The Control group had a greater accelerometer compliance than the Intervention group at week 8 and follow-up (Intervention: baseline, 64%; week 8, 24% and; follow-up, 14%. Control: baseline, 61%; week 8, 40% and follow-up, 26%).

Participants’ absence from school on days which data collection took place was the reason for missing health-related quality of life data and some participants abstained from the fitness tests assessment. Fifty children in the intervention group and 63 in the control group completed all physical fitness measures at week 8 and at follow-up. Of the children who completed the health-related fitness questionnaire at baseline, 23 children also completed at week 8 and follow-up for intervention condition and 54 in control.

### Outcomes measures

Table 3 details the mean total PA (cpm), mean MVPA (min/day), physical fitness, and health-related quality of life for both intervention and control at week 8 and follow-up.

**Table 3:** Adjusted mean outcome values for week 8 and follow-up.

Variable	Time Phase	INT*	CON*	Difference	95% Compatibility Interval^a^
**MVPA (min/day)**	Week 8	69.7	66.9	2.8	-12.5 to 18.0
	Follow-Up	73.9	66.9	7.0	-8.8 to 22.8
**Total PA (cpm)**	Week 8	661	663	-2	-127 to 124
	Follow-Up	683	672	11	-121 to 143
**Bal (no. of falls)**^**b**^	Week 8	5.6	8.1	-2.5	-4.3 to -0.75
	Follow-Up	5.9	7.2	-1.3	-3.1 to 0.5
**HECo (s)**	Week 8	139.8	144.6	-4.8	-16.0 to 6.3
	Follow-Up	138.7	146.4	-7.7	-18.9 to 3.5
**S&R(cm)**	Week 8	16.0	16.3	0.3	-5.7 to 5.0
	Follow-Up	17.0	18.7	-1.7	-7.1 to 3.6
**Broad Jump (cm)**	Week 8	132.0	136.0	-4.0	-17.3 to 9.4
	Follow-Up	134.2	140.4	-6.2	-19.6 to 7.3
**Sit Ups** **(no. completed)**^**b**^	Week 8	16.7	16.5	0.2	-2.4 to 2.9
	Follow-Up	15.7	16.7	-1.0	-3.6 to 1.7
**5m SR (s)**	Week 8	203.9	200.8	3.1	-15.2 to 21.5
	Follow-Up	203.5	211.1	-7.6	-26.0 to 10.8
**ERSpeed (km/h)**	Week 8	10.54	10.45	0.09	-0.22 to 0.40
	Follow-Up	10.30	10.56	-0.26	-0.58 to 0.05
**Phys WB**	Week 8	52.0	53.1	-1.1	-4.8 to 2.6
	Follow-Up	53.7	52.7	1.0	-2.7 to 4.7
**Psych WB**	Week 8	52.6	50.3	2.3	-2.5 to 7.2
	Follow-Up	52.1	52.5	-0.4	-5.3 to 4.5
**Autonomy & PR**	Week 8	48.0	50.1	-2.1	-6.6 to 2.3
	Follow-Up	55.1	52.8	2.3	-2.1 to 6.7
**Peer & SS**	Week 8	55.8	50.5	5.3	0.6 to 10.0
	Follow-Up	56.2	51.5	4.7	-0.1 to 9.4
**School Enviro**	Week 8	55.4	55.7	-0.3	-4.3 to 3.6
	Follow-Up	56.2	55.5	0.7	-3.3 to 4.6

CON = control group. INT = intervention group. Total MVPA = total moderate-to-vigorous physical activity. Total PA = daily total physical activity. Bal = balance. HECo = hand eye coordination. S&R = sit and reach. 5mSR = 5m shuttle run. ERSpeed = effective running speed. Phys WB = physical wellbeing. Psycho WB = psychological wellbeing. Autonomy & PR–autonomy and parent relations. Peer & SS = peer and social support. School Enviro = school environment.

*The mean for CON represents the effect in an average (typical) cluster that does not receive the intervention, with the mean for INT giving the effect in an average cluster undertaking the intervention.

^a^The compatibility interval represents a posterior distribution for the primary physical activity outcomes.

^b^Strictly, these variables are count outcomes, but the linear mixed model produced well behaved residuals for these and all other outcomes.

The posterior probability of a beneficial effect of at least 5 min/day for MVPA at 8 weeks was 39% (possibly beneficial). At 4 weeks post-intervention the probability of benefit was 60% (possibly beneficial). Note, however, from the 95% compatibility intervals presented in Table 3 that substantially negative effects (worse than control by >5 min/day) are also reasonably compatible with the data and model, indicating that the results are inconclusive. The mean effects on total physical activity were negligible, again with wide compatibility limits.

### Structured discussion & focus groups

Two structured discussions were conducted with the teachers who taught in the intervention schools (structured discussion 1, n = 2; structured discussion 2, n = 3). The main author led the discussion with a question schedule used as a guide to facilitate. The questions covered the acceptability and sustainability of the ExCiTE activities. Focus groups were conducted with participating children in each intervention school. Nine focus groups were conducted with five or six children per focus group, totalling 50 children and an equal distribution between the two intervention schools. Questions covered children’s enjoyment and acceptability of the intervention. The key themes from the teacher structured discussions are detailed in Table 4 and the key themes for the child focus group are detailed in Table 5.

**Table 4:** Key themes on acceptability and sustainability of the ExCiTE intervention from teacher structured discussions.

Theme	Quote
Delivery	“I liked delivering them from the reaction from the kids” “I tended to focus on times table activities” “Those activities that needed some preparation beforehand were difficult” “It was easy to work with something like times tables because it is repetition that they need so that worked well” “Once you were familiar with it and you knew they enjoyed that activity then why change it–they were requesting the same ones a lot”
Engagement	“There was the odd one that was doing it off beat just to be funny…then you have those that kind of stood back”
Enjoyment	“I liked them because they were different…they were more physically challenging and it was good you can link them together” “I was daunted by them …the kids really liked them though…after you get going with it then it is alright”
Classroom Behaviour	“My class are quite immature, I thought there would be silliness, but I was genuinely surprised with how well behaved they were” “They were just trying to show off and be a little silly…it was just a few boys” “Some boys were showing off, but they weren’t being naughty”
Sustainability	“I have taken all my stuff to Key Stage 1 and I have picked the ones out that I am going to start with in September” “I would implement it within my lessons, and most activities I would do, but I wouldn’t do the activities that needed prep” “I would love to continue delivering them but, if I am honest, I don’t know for how long that would be because it is just constraints unless it became part of the school-day curriculum and it was obligatory”
Confidence	“I don’t think I was confident enough” “I would like to try a wider range of activities next year now I am more familiar”

The key themes emerging from the structured discussions with teachers were; delivery pattern, engagement and enjoyment of the children, classroom behaviour and management, sustainability of the intervention and the confidence levels of the teacher. The teachers tended to deliver the same activities, and these were the activities that required less preparation, possibly due to a lack of confidence. The teachers reported that the Mathematics activities were easier to deliver, which could explain why teacher favoured delivering these activities. The teachers’ lack of confidence also appeared to impact on their choice of activity to deliver, with teachers opting to deliver activities they were familiar with and perceived as easy. The teachers found the children engaged well with the activities and they did not appear to impact on behaviour. The teachers suggested they would deliver the activities again to different year groups; however they suggested that for effective implementation a “whole-school” approach would likely be needed.

**Table 5:** Key themes on acceptability and sustainability of the ExCiTE intervention from child focus groups.

Theme	Quote
Enjoyment	“you get to move around and it is fun” “it is fun because you get to do stuff you haven’t done before” “sometimes the times tables is boring but this made it fun” “sometimes it was hard to act out” “I struggled to keep in time with the jumping” “it got boring when we did the same activities”
Fitness	“it helps people get fit” “everyone was puffed at the end”
Perception of Learning	“I think it is an easier way to learn because it is fun” “the spelling and literacy helped me with the exams–I could remember jumping around” “it is like you are enjoying learning, like most other lessons are boring….I got excited to do the activities”
Transition to class work	“I felt energetic to work” “you were calmed down after the activities because you were exhausted” “before the activities you felt like you were going to fall asleep, this woke you up” “when you first sit down you are thinking about it but it gets you puffed out so you are glad to get on with your work” “we just go straight back to our work, that makes our teacher happy” “when you were bored, you wanted to do it again” “I found it hard to concentrate after bouncing around… like your mind is still thinking about what you have been doing”
Delivery	“we didn’t do them every day” “it would be good to do different activities”
Teacher Involvement	“it was good when the teacher got involved….. joining in with us” “she didn’t always do it with us, she just read stuff out and watched us” “She was lazy and just wanted to sit down and eat her chocolate cake. She should do it then it would show us that she is fit” “He was lazy sometimes”
Changes to the ExCiTE intervention	“Get the teacher to join in” “Link the activities more to our lesson, like you’d be talking about space then do an activity to connect them” “Do them more often” “It would be more fun if we could do it in groups” “Nothing, they were the best activities we have ever done!” “Maybe you could do a video so we can see how it is done” “Do different activities, I know there were quite a lot in the book, but we only did the same ones”

The themes emerging from the children’s focus groups were similar to those from the structured interviews with the teacher, with children reporting on enjoyment and delivery. However, the themes that emerged that were different to those of the teachers were; perception of learning, awareness of fitness, transition between PA and classroom work and teacher involvement. Most of the children did not appear to associate the activities with learning and they felt energised following the activities. The children disassociated the EXCiTE activities with learning, feeling as though it was a break from learning; only when explored further, prospectively, did the children realise they were learning. Some children were able to focus on class work following the activity; however, some found the transition difficult, citing difficulties in concentration. Most children felt the activities made them work hard and were enjoyable than usual class work, liking the novelty of the activities. The children expressed a strong desire for the teachers to join in the activities expressing negative feelings towards the lack of engagement by the teacher, perceiving the teacher as lazy. The children provided some interesting suggestions for changes to the intervention, including the use of videos, more variety of activities, linking them more explicitly to the curriculum subject, more teacher involvement and working in groups.

## Discussion

The current study was a small non-randomised exploratory trial, and the effect of the curriculum-embedded and classroom-based ExCiTE intervention on MVPA versus control at post-intervention and at follow up was inconclusive. The uncertainty in the estimate showed that substantial negative effects, trivial effects, and substantial positive effects were reasonably compatible with the data and model. Previous classroom-based PA studies have shown that incorporating PA into the curriculum can increase step count, energy expenditure, and total PA [13, 34, 35]. The ExCiTE intervention was enjoyed by teachers and children and the schoolteachers reported continuation of the activities.

The school setting provides several opportunities to intervene with children, with evidence showing that school-based interventions have a positive effect on PA [3]. However, schools are a complex ecological system in which many constituent components interact with behaviour, and where flexibility in tailoring the intervention is required for those delivering or receiving the intervention [36]. Since PA school-based interventions are events implemented in a dynamic and complex system, it has been theorised that longer time frames for follow up are required as changes might not be linear, and a better understanding of pre-intervention context might be required [37]. In this study, intervention and control schools had similar characteristics concerning number of children attending, socioeconomic status, and physical education provision (Table 1). However, control schools presented a substantially higher mean MVPA and total PA at baseline (Table 2) and had greater accelerometer compliance across all testing phases, which highlights some differences in the pre-intervention context.

The fitness test results are purely exploratory and descriptive, but there was little indication of substantial differences between the intervention and control groups at week 8 or follow-up. To our knowledge, only one other study has measured the effect of a classroom and curriculum-based PA intervention (ABC for fitness) on fitness components [38]. The ABC for fitness intervention improved upper body strength, abdominal strength, and trunk extension compared to control. However, the study was completed over a school year (September to April); therefore the time scale of the current study may not have been large enough to affect physical fitness. Likewise, the duration and differences in the delivery of the ExCiTE activities by the teachers might have affected some of the physical fitness results. Nevertheless, a recent review found no effects on fitness outcomes [39].

Results from the KIDSCREEN questionnaire–again descriptive and exploratory—revealed that the intervention might have a small beneficial association with ‘peers and social support’ at week 8 and at follow-up (Table 2). This association would be congruent with the group-orientated nature of the ExCiTE intervention, and is worthy of future investigation. Nevertheless, the number of complete questionnaires was limited due to missing responses or inaccuracies (i.e. ticking all boxes to the questions). To alleviate some of these issues, using digital technology (i.e. online questionnaire) might be advantageous.

This study is one of the few which examined the views of teachers in relation to classroom-based interventions [40]. Enjoyment from the teachers and children is crucial for the delivery, acceptance, and sustainability of PA interventions. The teachers felt the activities were enjoyed by the children, linked appropriately with the curriculum and would be beneficial as a whole-school scheme for sustainability and continuity throughout the years. The teachers did report a lack of confidence in delivering some of the activities, which resulted in their primarily delivering a select number of activities that required less physical skill and planning. Implementation was explored in terms of the number of ExCiTE sessions reported by the teachers. Although the perceived intensity and frequency of the activities were obtained from the focus group discussions these variables were not measured directly. Future research could employ robust methods to monitor the intensity of the intervention, such as heart rate monitoring. The teachers were asked to deliver a 10-min ExCiTE activity each day, but they reported delivering the intervention on average three times a week. This reported frequency is similar to other studies [14, 17] but greater than some reported interventions were implemented only once a week [16].

The children reported that they liked the activities but noted the repetitious nature and requested more variety. However, the teachers opted to deliver the same activities from the pack due to the lack of confidence and time-constraints; therefore, the teacher selected an activity that required less preparation and that they were familiar with. Teachers requesting “ready to use” activities that require little preparation has been noted in previous research [41]. The lack of variety expressed by the children has also been shown in previous research (42) indicating children became ‘bored’ by the repetition of an activity. The lack of confidence of the teacher to deliver physically active lessons has been previously reported and it appears to be a crucial element to the sustainability of classroom-based PA interventions [42]. Like previous findings, the children enjoyed the opportunity to move in the classroom and they perceived the activities to be a break from learning [43]. However, the children reported that they would prefer the teachers to have more involvement in the activities.

Although not measured directly, the children reported that they were able to remember the subject content delivered in the ExCiTE activity clearly. However, the children only reported the subject content of the activity, and not the content following the activity. This observation therefore questions whether curriculum-based PA breaks improve memory and potentially academic performance in the long-term or only at an acute stage. The current literature on the association between PA and academic performance and PA is equivocal. However, most studies suggest that increase in PA does not negatively affect academic performance [44, 45], although it appears that cardiorespiratory fitness could be a marker of academic performance [46]. In relation to subject area, the children recalled Mathematics ExCiTE activities more often. This observation is worthy of future exploration, as evidence suggests that PA may improve numeracy in some children [45, 47]. However, the teachers reported that they delivered more numeracy ExCiTE activities which could explain the children’s responses. Therefore, further research on how the ExCiTE activities impact on academic performance in different subject areas is required.

This study has several strengths including the mixed-methods design, the use of objective measures of PA, the assessment of physical fitness and health–related quality of life, and the inclusion of follow up measures. One of the limitations of this study was the low compliance to accelerometer wear for both groups at post-intervention and follow up. Although we applied a principled analysis approach for addressing missing data, the proportion missing is very high at these timepoints. Compliance decreased substantially across the testing phases. At baseline, the proportion of our sample providing at least 4 valid days was higher than that reported in a major national survey for the equivalent age group [48]. The lead author implemented several evidence-based suggestions to increase compliance, such as rewards for returning monitors, and regular contact with the teachers and children [49]. Also, the ExCiTE activities were not consistently performed daily for the 8-weeks, as planned. Schools are dynamic and changeable environments with conflicting agendas. A whole-school approach, with activities embedded into the curriculum and school policy, might be required for future studies to ensure daily delivery. The focus groups and structured interviews were conducted by the lead researcher which could have led to socially desirable answers, meaning the teacher and schoolchildren give more favourable responses to the questions. The study design altered from a planned cluster randomised controlled trial to a non-randomised design due to strong school preference and non-consent to randomization. There might have been a more positive attitude towards physical activity and fitness in schools that opted to be in the intervention arm, which might have increased the teacher engagement with the intervention and influenced the findings in unknown ways. Finally, this is an exploratory, non-randomised study and no robust causal inferences may be made. There was a very small number of clusters (n = 5) for the random effect, reducing power and precision, and the results of the mixed model analysis should be interpreted with caution. Nevertheless, this study still provides valuable information to inform future studies. A large, properly powered cluster randomised trial is required to evaluate the effectiveness of classroom-based, curriculum-embedded PA interventions.

## Conclusion

Classroom-based, curriculum-embedded PA appears to be a feasible approach to adopt PA in the school environment. In the current study, the effect of the ExCiTE intervention on average daily MVPA at both post-intervention and follow-up timepoints was inconclusive. The qualitative component of this study shows that, overall, the ExCiTE intervention was received positively by the teachers and children. The teachers reported that the intervention could be applied into practice effectively and sustainably with minor adjustments. Future developments could include: providing digital format of the activities to support the teachers’ delivery of the activities, and a more in-depth teacher training to build their confidence. A properly powered, cluster RCT with a longer intervention, in different socioeconomic areas is required. Also, future studies need to consider methods to increase accelerometer compliance, especially for follow-up measures, or to use a device associated with better compliance such as a wrist-mounted unit. The ExCiTE intervention would benefit from including an objective assessment of learning, or at least a proxy for learning such as on-task behaviour to determine the impact on academic achievement.

## Supporting information

S1 File Outcome data. (XLSX)

S2 File Populated TREND statement checklist. (DOCX)

S3 File Study protocol. (DOCX)

S4 File ExCiTE Activity example. (DOC)
