# Supplementary material for: The effect of a curriculum-based physical activity intervention on accelerometer-assessed physical activity in schoolchildren: A non-randomised mixed methods controlled before-and-after study
Source: PLoS One. 2019 Dec 5;14(12):e0225997. doi: 10.1371/journal.pone.0225997 (PMC6894866; doi:10.1371/journal.pone.0225997)
Supplement: S3 File — (DOCX) [file pone.0225997.s003.docx]

**Ethics Protocol**

**Introduction**

The school is an obvious setting to educate and promote physical activity as children spent the majority of their waking day at school and all children have a right to attend public school in the UK (Fox *et al*. 2004). However, schools are under pressure from government officials to meet academic standards which often results in reduced physical activity and physical education time. A new phenomenon to combat the problem is to incorporate physical activity into the curriculum. Although anecdotal evidence suggests that incorporating physical activity in the classroom is not a new phenomenon among UK primary school teachers, these classroom-based strategies have not been evaluated in the literature. However, in America there are a few evaluations of interventions such as Physical Activity Across the Curriculum (PAAC) (Honas *et al.* 2008), Energisers (Mahar *et al*. 2006) and Take 10! (Stewart *et al.* 2004) which suggest that classroom-based strategies may promote physical activity and academic achievement in school children (Reed, 2009). The primary method used to assess physical activity in the current literature is pedometers, which are inaccurate to determine energy expenditure (Bassett, 2000) and they do not measure intensity, duration or frequency of physical activity (Andre & Wolf, 2007). Also, there is a lack of randomized controlled trials in order to investigate whether physical activity during school hours affects physical activity after school. Based on the current limitations within the research, the aim of the exploratory, randomised controlled trial is to;

1. Determine the influence of a classroom-based physical activity intervention incorporating the curriculum on total physical activity.
2. Examine the effects of a classroom-based physical activity intervention incorporating the curriculum on health-related quality of life, dietary intake, classroom behaviour and fitness.
3. To gain an understanding of the experiences of, and views and attitudes towards the intervention among participating children and the school teachers.

**Methods**

**Participants**

4-6 clusters of comparable primary schools from Middlesbrough and the surrounding region will be approached to take part in the school. The schools will be recruited and allocated to either an Intervention arm or Control arm and will be matched according to Index of Multiple Deprivation, size and provision of physical education. Year 5 and 6 school children (aged 9-11 years) will be invited to take part in the study. Those who provide parental/guardian informed consent and child assent will be included in the study. The study aims to recruit a minimum of 100 participants (N=50 intervention and N=50 control group) with an equal gender mix in order to represent the general population.

**Study Design**

The intervention group will complete EXCITE activities, daily for 8-10 weeks. EXCITE activities are based on a curriculum subject (2=English, 2=Mathematics, 2=Science) and involve physical movement such as jogging, marching and jumping. Following the 10 minute activity the teacher will deliver a 2-3 minute cool down consisting of light exercise and stretching to lower heart rate then the participants will continue their usual school routine. The control group will continue with their usual school routine. Measurements will be taken at baseline, after the intervention and at follow-up.

**Measurements**

All measures will be conducted at the school. The following outcome measures will be taken at Baseline, Post-Intervention and Follow-up.

**Height and Weight**

All children will have their height and weight measured at the school using a stadiometer and a scale respectively. The participant will need to take off the shoes and stand on the scale while the researcher measure weight and height.

**Total Physical Activity**

The participant will wear two small pieces of equipment; an accelerometer (ActiGraph GT3X) and a heart rate and movement monitor (ActiHeart) to measure physical activity. The ActiGraph will be worn on an elastic belt around the hip. The ActiHeart will be secured to the skin by two sticky electrodes on the chest. The ActiHeart will be fitted in private room by the researchers and there will always be a second adult person in the room. The participant will wear the monitors for waking hours for 8 days. The participants will also be asked to complete a physical activity diary. The participants will be given instructions about the monitors.

**ActiHeart: Step test**

To increase the estimation of physical activity intensity by means of the Actiheart measurement an individual calibration is required to account for the individual variability on heart rate and physical activity level (Rennie *et* *al*. 2001). This test will also give an estimation of the maximal oxygen uptake (VO_2_ max). The trial will consist of 8 min step test (Brage *et al*. 2007). Following an audible metronome, the test will start at 15 body lifts/min and progressively increase the 33 steps per minute on an 18.5-21.5 cm high step. Recovery heart rate will be recorded for 2 min after the completion of the test.

The Actiheart will be set at 32Hz for accelerometer data and 128Hz for heart rate data. Data will be recorded in 5-s epoch during the step test. The Actiheart will be attached on the participant skin by two electrocardiogram (ECG) electrodes. Before electrode placement the skin will be prepared. Skin preparation involves cleaning the skin and then, through gentle abrasion remove the layer of dead skin – the *stratum corneum*. The most effective and practical method is very gentle abrasion using the purpose-made Cardioprep® pads, which results in minimal skin damage or irritation. A few gentle strokes decrease impedance substantially with little or no skin reddening.

The Actiheart accelerometer will clip onto a ECG electrode with a lead to another electrode that picks the ECG signal. The electrodes will be placed on the left side of the chest. The medial electrode will be placed midway between V1 (fourth intercostal space to the right of the sternum) and V2 (fourth intercostal space to the left of the sternum). The other electrode will be placed at V4 (fifth intercostal space at midclavicular line) or V5 (level with V4 at left anterior axillary line). The medial and lateral electrodes have to be placed at the same horizontal level to avoid reading error. The cable should also be stretched to its full length to avoid unnecessary rotation of the sensor.

**Dietary Intake**

The participants will completed the Food Intake Questionnaire (Johnson *et al.* 2001)

**Health –Related Quality of Life**

The participants will be asked to complete the 27-item Kidscreen Questionnaire ([Ravens-Sieberer](https://springerlink3.metapress.com/content/?Author=Ulrike+Ravens-Sieberer), *et al*. 2007)

**Classroom Behaviour**

The school teacher will be asked to complete the Teacher Version of the Strength and Difficulties Questionnaire (Goodman, 1997) for each child involved in the study.

**Physical Fitness**

At the school the children will complete a series of fun, validated child-relevant fitness tests (e.g. push-ups, sit-ups, flexibility exercises, aerobic endurance and fundamental motor skills)

*Intervention Group Only*

**Focus Group with Children**

Following the intervention, those children involved in the Intervention Group will be given the opportunity to participate in a focus group to express their views and opinions of the intervention. Each focus group will last approximately 30 minutes and will take place at the child’s primary school. At the start of the focus group the participants will be informed both written and verbally the aims of the focus groups. Both parent/guardian informed consent and child assent will be gained prior to the focus group. The participants will be made aware that there are no right or wrong answers, they will be told to respect the opinions of others and that all focus groups will be audio-taped.

**Focus Group with Teachers**

The teachers will also be offered the opportunity to attend a focus group following the intervention to express their thoughts and feelings about the intervention. The focus groups will follow the same format as the child focus groups but these will be approximately 1 hour.

**References**

Andre, D., & Wolf, D.L (2007) Recent Advances in Free-Living Physical Activity Monitoring: A Review. *Journal of Diabetes Science and Technology*, **1(5),** 760-767.

Bassett, D.R (2000) Validity and reliability issues in objective monitoring of physical activity. *Research Quarterly Exercise and Sport*, **71**, 30-36.

Brage, S., Ekelund, U., Brage, N., Hennings, M. A., Froberg, K., Franks, P. W., & Wareham, N. J (2007) Hierarchy of individual calibration levels for heart rate and accelerometry to measure physical activity. *Journal of Applied Physiology*, **103**, 682-692

Fox, K.R., Cooper, A., & McKenna, J (2004) The school and promotion of children’s health-enhancing physical activity: perspectives from the United Kingdom. *Journal of School Health*, **23,** 338-358

Goodman, R. (1997) The Strengths and Difficulties Questionnaire: A Research Note. *Journal of Child Psychology and Psychiatry*, **38**, 581-586.

Honas, J.J., Washburn, R.A., Smith, B.K., Greene, J.L. & Donnelly, J.E. (2008) Energy Expenditure of the Physical Activity across the Curriculum Intervention. *Medicine & Science in Sports & Exercise,* **40 (8),** 1501-1505.

Léger, L.A., Mercier, D., Gadoury, C., & Lambert, J. *(1988)* [The multistage 20 metre shuttle run test for aerobic fitness](http://www.informaworld.com/smpp/content~db=all~content=a785360684~frm=titlelink). *Journal of Sports Sciences*, **6 (2),** 93-101.

Mahar, M.T., Murphy, S.K., Rowe, D.A., Golden, J., Shields. A.T. & Raedeke, T.D. (2006) Effects of a Classroom-Based Program on Physical Activity and On-Task Behavior. *Medicine & Science in Sports & Exercise*, **38(12),** 2086-2094.

# [Ravens-Sieberer](https://springerlink3.metapress.com/content/?Author=Ulrike+Ravens-Sieberer), U., [Auquier](https://springerlink3.metapress.com/content/?Author=Pascal+Auquier), P, [Erhart](https://springerlink3.metapress.com/content/?Author=Michael+Erhart), M., [Gosch](https://springerlink3.metapress.com/content/?Author=Angela+Gosch), A., [Rajmil](https://springerlink3.metapress.com/content/?Author=Luis+Rajmil), L., [Bruil](https://springerlink3.metapress.com/content/?Author=Jeanet+Bruil),J., [Power](https://springerlink3.metapress.com/content/?Author=Mick+Power), M., [Duer](https://springerlink3.metapress.com/content/?Author=Wolfgang+Duer), W., [Cloetta](https://springerlink3.metapress.com/content/?Author=Bernhard+Cloetta), B., & Czemy, L. (2007) The KIDSCREEN-27 quality of life measure for children and adolescents: psychometric results from a cross-cultural survey in 13 European countries. *Quality of Life Research*, 16(8), 1347-1356.

Reed, J (2009) *Active Education; Lessons for Integrating Physical Activity with Language Arts, Math, Science and Social Studies*, Nova Science Publishers, Inc.

Rennie, K. L., Hennings, S. J., Mitchell, J., & Wareham, N. J (2001) Estimating energy expenditure by heart-rate monitoring without individual calibration. *Medicine & Science in Sports & Exercise,* **33**, 939-945

Stewart, J.A., Dennison, D.A., Kohl, H.W. & Doyle, A.J. (2004) Exercise Level and Energy Expenditure in the Take 10! In-Class Physical Activity Program. *Journal of School Health*, **74 (10)**, 397-400).
