# Supplementary material for: The effect of a curriculum-based physical activity intervention on accelerometer-assessed physical activity in schoolchildren: A non-randomised mixed methods controlled before-and-after study
Source: PLoS One. 2019 Dec 5;14(12):e0225997. doi: 10.1371/journal.pone.0225997 (PMC6894866; doi:10.1371/journal.pone.0225997)
Supplement: S4 File — (DOC) [file pone.0225997.s004.doc]

**EXCITE ACTIVITES**

**MATHEMATICS**

**Structure**

Choose an EXCITE activity (remember you can adapt the activities)

Deliver the activity for approximately 10 minutes

Perform a cool down for 2-3 minutes

**Preparation Required**  **Intensity Level**

1

2

3

None/little needed

Some needed

Most needed

**
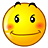
**

**
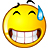
**

Low/light

Moderate

Vigorous/tiring

**
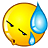
**


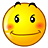
**Jumping Jack Maths**

1

**Purpose**

**To be complete maths problems without pen and paper.**

**Time**: 10 minutes, followed by 2-3 minutes cool down

**Equipment**: Maths problems

**Formation:** standing at desks

**Instructions**

1. Start = all students jogging or marching on the spot
2. Teacher/Lead calls out a maths problem (for example addition, subtraction, multiplication, or division).
3. Student must mentally solve the problem and perform the corresponding number of jumping jacks.

**Easier**

- Students step out the jumping jacks (less impact)

**Harder**

- Jumping star jumps

**Variation**

- Incorporate more complex maths problems
- Add different movements
- Students could work in pairs or small groups to give the answers (jumps) if choosing large numbers
